# Increasing engagement in professional governance from the unit to system level

**DOI:** 10.1097/nmg.0000000000000245

**Published:** 2025-04-29

**Authors:** Tanya F. Lott, Alison Partridge

**Affiliations:** At Roper St. Francis Healthcare in Charleston, S.C., **Tanya F. Lott** is the director of Nursing Excellence and **Alison Partridge** is a research nurse scientist.

**Figure:**
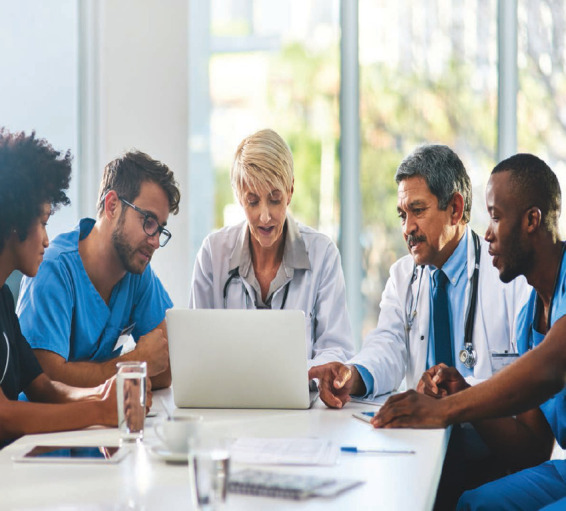
No caption available.

The positive benefits of shared decision-making in nursing are well documented in the literature.^1^,^2^ A four-hospital healthcare system had witnessed these benefits firsthand in the growth of its nurses' professional development and engagement in decision-making, leading to improved patient outcomes. Established in 2007 at the system's two flagship hospitals, shared governance was engrained as part of the nursing culture; councils were even implemented within the first 6 months of opening two brand-new hospitals in 2012 and 2019. As the organization experienced increased nurse turnover related to the pandemic and the nursing shortage, engagement in the organization's shared decision-making structure had steadily declined by the end of 2021.

A Nursing Excellence department was established in 2021 at the system level to lead all hospitals in nursing excellence designation journeys, including oversight of shared governance. Prior to 2021, each hospital within the system had its own shared governance structures and processes. The system consisted of one three-time American Nurses Credentialing Center (ANCC) Magnet®-recognized hospital and one three-time ANCC Pathway to Excellence®-designated hospital at the time when the Nursing Excellence department was established. One purpose of this new team was to standardize and strengthen shared decision-making across the healthcare system to increase engagement and improve efficiency.

## PLANNING

**FIGURE 1:**
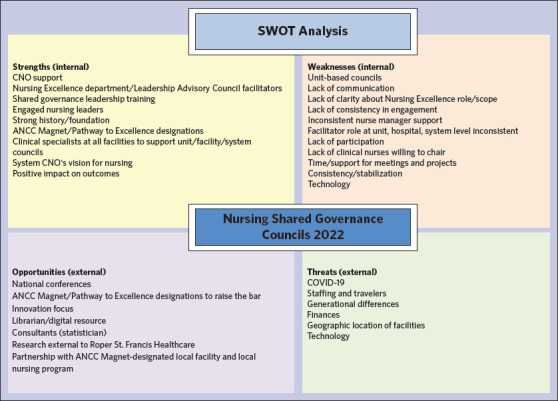
SWOT analysis of existing shared governance structure

The first steps that the Nursing Excellence department completed were an assessment of the current shared governance structure and a synthesis of the current literature. The team chose Lean Six Sigma as the project management framework to standardize the organization's shared decision-making processes, which would enhance efficiency and outcomes. The department met in January 2022 to complete a SWOT (strengths, weaknesses, opportunities, threats) analysis of the current structure (see Figure 1). Many strengths were noted; however, the weaknesses and opportunities identified included a decrease in active unit-based councils, a decrease in engagement and clinical nurse leaders, confusion about roles and purposes, the impact of COVID-19 and the nursing shortage, and other inconsistencies across the healthcare system.

### Literature synthesis

In their integrative review, Kanninen and colleagues identified professional governance as a means to improve nurses' professionalism by creating a more engaging work environment.^3^ Interventions found to strengthen shared decision-making in hospitals that were incorporated into the process improvement project included evaluating existing structures and creating new structures where needed, enhancing teamwork through unit councils, and building leadership skills through professional governance training.^3^ Evidence identifies the impact nurse managers have on setting the tone of nursing units and supporting shared decision-making engagement.^3^,^4^ This concept was incorporated into the project plan by first engaging with nursing leaders and then establishing new structures and processes.

O'Grady and Clavelle found that participation in councils doesn't equate to ownership of decisions and actions.^5^ Measuring the impact of shared governance historically has focused on what exists within a shared governance structure instead of a focus on the behaviors of the individual nurse.^6^ The change to professional governance places the focus on nurses' responsibility to self-govern the practice of nursing and the social agreement that nurses must continuously improve nursing care.^5^,^6^ This concept was the rationale for changing from a shared to professional governance model.

### Lean Six Sigma project charter

The project charter consisted of the following elements.

***Problem statement***. There's a lack of standardization of shared governance councils across the healthcare system.

***Big hairy audacious goal (BHAG)***. Every nurse will have the same opportunity to be fully engaged in all levels of shared governance no matter the unit, department, or hospital in which they work.

***Project mission statement***. Enhance the engagement of nurses in the shared decision-making process at all levels (unit, hospital, and system).

***Project scope***.

- Inclusion: bylaws, structures/process/outcomes shared governance, shared governance leadership role development
- Exclusion: professional practice models, care delivery systems, scope of nursing care (these would remain unchanged)

***Stakeholders***. Clinical nurses, clinical specialists, nurse managers, directors, CNOs, vice president of nursing.

***Business case***. Standardization will lead to increased RN engagement, patient satisfaction, and nursing quality outcomes.

### Voice of the customer

To ensure the customer's voice was included in the project, the Nursing Excellence department conducted a survey via email of nursing leadership and current shared governance members to assess their views on the current structures and potential changes. Questions were based on a 5-point Likert-type scale, ranging from 1 (least effective) to 5 (most effective).

Sixty-one nurses completed the survey. The nurses identified that the current shared governance structure was most effective in ensuring nursing practice was up to date and improving nurses' professional growth and development (see Table 1). The areas in which shared governance was currently least effective were improving RN retention and improving nurse well-being/mental health. The survey consisted of several open-ended questions to capture respondents' ideas and provide an opportunity for full feedback. Examples of feedback included ideas to improve participation, increase the councils' focus on actions and outcomes, provide an opportunity for nurses to look beyond the current COVID-19 space to nursing as a whole, and incorporate more technology into the councils. Most respondents (89%) agreed to change the current structure of shared governance, and 80% supported the transition from shared to professional governance.

**TABLE 1:** Voice of the customer survey during preplanning phase (n = 61 shared governance participants and nursing leaders from across the system)

Effectiveness of shared governance question	Mean	SD
Positively contributing to nurse satisfaction	3.12	1.17
Improving nurses' professional growth and development	3.18	1.27
Ensuring nursing practice is up-to-date	3.23	1.20
Contributing to nursing research	2.98	1.27
Improving nursing-sensitive quality indicators and patient satisfaction	2.95	1.31
Improving RN retention	2.38	1.28
Improving nurse well-being/mental health	2.57	1.24

### Overall plan

The overall project plan included first focusing on unit-based councils in 2022 and implementing a new systemwide structure in 2023. Unit-based councils were identified as the current greatest gap in the structure; therefore, the Nursing Excellence team decided to begin improvement efforts with these councils while creating the new system structure for full transition from shared to professional governance.

## INTERVENTIONS/IMPLEMENTATION

### Unit-based council development and enhancement

The first goals of the project were to create councils that didn't exist currently on nursing units and to enhance councils that already existed. Recognizing the impact nursing leadership support has on shared decision-making, the team's first step toward accomplishing these goals was to create education for the nursing clinical managers, directors, and CNOs and present this information at each hospital's nursing operations meeting. The education focused on the purpose of unit-based councils and the essential role nurse managers have in the success of these councils.

As the team developed a new model and structure, unit councils were moved to the epicenter of the model, emphasizing the unit councils' integral part in a strong, successful shared decision-making structure. The purpose of the unit-based council is to enable two-way communication for input into decisions happening at the hospital- and system-level councils. Unit-based councils also address unit-specific issues and implement the decisions made at system- and hospital-level councils.

**FIGURE 2:**
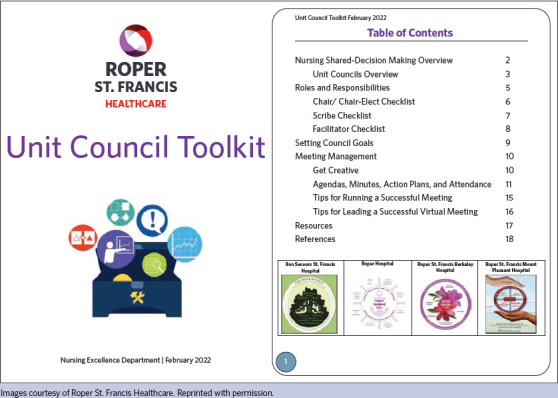
Unit Council Toolkit cover and table of contents

To support the development and enhancement of unit-based councils across the healthcare system, the Nursing Excellence department created a Unit Council Toolkit (see Figure 2). The tool kit included an overview of shared governance and its benefits, the role of the clinical manager, ideas for council activities (because not knowing what specifically to focus on was often cited as a barrier to starting a council), roles and responsibilities with leader checklists to simplify the expectations, goal setting, meeting management (including templates and tips for meeting minutes and agenda setting), and tips for running an effective meeting. The team also added tips for conducting a successful virtual meeting because many councils had started to meet virtually.

The tool kit was printed and delivered to each nurse manager during the nursing operations meetings in February 2022. Additional education and information were shared at the July 2022 nursing operations meetings to ensure the focus remained on enhancing unit councils. At these July meetings, nurse managers who had effective councils were asked to share what was working and how they made the unit-based council a priority.

To further guide nurse leaders in creating strong unit councils, the Nursing Excellence program managers met with nurse managers one-on-one to provide direction and assistance. They also hosted several drop-ins on units where nurses could stop by and learn more about shared governance and the plans for a unit council. These sessions were used to generate excitement and answer any questions before councils were officially launched or relaunched. Upon request, nurse managers could also have another nurse manager mentor assigned to provide additional support and share what worked well for them in establishing a unit-based council.

As 2022 progressed, units implemented councils, and the momentum grew. During this time, the Nursing Excellence department created a new shared decision-making structure and gathered input from leaders and clinical nurses.

### Professional governance system structure and process implementation

On December 7, 2022, the Nursing Excellence department hosted its annual Shared Governance Leadership Workshop. Although this workshop had been in place to support and train council leaders since 2009, this was the first time that council leaders from across the system completed the training together.^7^ One purpose of the training was to introduce the transition from shared governance to professional governance and unveil the new model and structure.

Following a presentation on the current state of nursing by the system vice president and CNO, the director of nursing excellence provided education on shared decision-making and the differences between shared and professional governance. Shared governance had focused on the work of the councils and the structures created, but the shift to professional governance would place the focus more on the individual's accountability and professional responsibility.^4^ Professional governance consists of accountability, professional obligation, collateral relationships, and effective decision-making.^5^ Shared governance had brought nurses to the table; professional governance would require ownership and personal and professional accountability.

The system created its definition of professional governance that day, which was, “The purpose of Roper St. Francis Healthcare Nursing Professional Governance is to engage clinical nurses, leaders, and interprofessional colleagues in collaborative decision-making and action-driven initiatives related to the professional obligations of competence, knowledge, quality, and practice and to the enhancement of the work environment through professional advancement and retention.”

**FIGURE 3:**
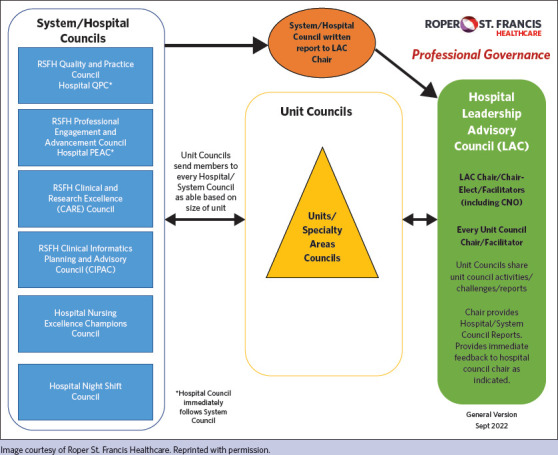
Professional governance structure

**FIGURE 4:**
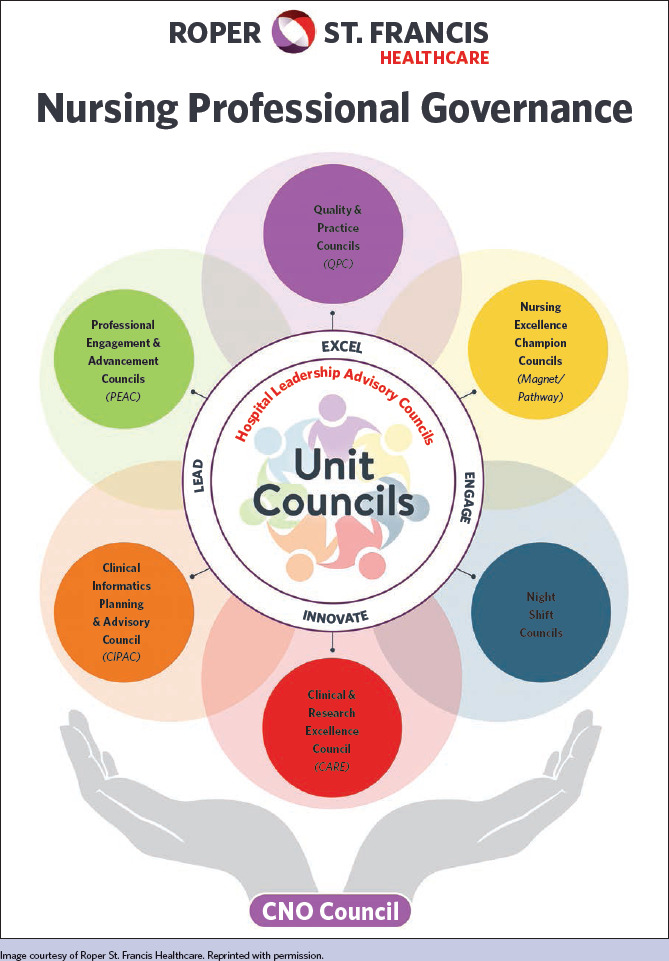
Nursing professional governance model

The new professional governance structure was also presented to this group, which included system-, hospital-, and unit-level councils (see Figure 3). The primary purposes driving the change in structure were to engage more clinical nurses and to improve the efficiency and effectiveness of the councils. The attendees voted on the new Professional Governance model as well (see Figure 4).

### New professional governance structure

Building upon the work to improve unit councils in 2022, the hospital and system councils were standardized across all four hospitals in 2023, and one set of bylaws was created that outlined each council's purpose and responsibilities.

Hospital-level councils included the following.

***Quality and Practice Council***. The purpose is to provide coordination and approval of practice changes that impact nursing and to continuously work to improve the quality of nursing care provided to patients.

***Professional Engagement and Advancement Council***. The purpose is to foster professional growth and development across the nursing spectrum and improve nurse retention through increased engagement.

***Nursing Excellence Champions Council***. The purpose is to increase awareness of the ANCC Magnet and Pathway to Excellence (Magnet/Pathway) standards, the impact and importance of Magnet/Pathway designation for the organization, and the benefits of being a Magnet/Pathway-designated hospital. It's a hospital-level council only.

***Night Shift Council***. The purpose is to provide a voice for the night-shift clinical nurses and healthcare team. It's a hospital-level council only.

***Leadership Advisory Council***. The purpose is to serve as the hospital's coordinating council, providing oversight and support for all council activities and leadership. It's composed of the chair, chair-elect, and facilitator of every unit, hospital, and system council.

System-level councils included the following.

***Quality and Practice Council***. Includes the four hospital councils and other system members.

***Professional Engagement and Advancement Council***. Includes the four hospital councils and other system members.

***Clinical and Research Excellence Council***. The purpose is to cultivate continuous clinical excellence through the promotion of clinical inquiry, evidence-based practice, and research to improve care delivery and patient outcomes. Its responsibilities don't require a hospital-level council; therefore, it's a system-level council only with representation from all hospitals.

***Clinical Informatics Planning and Advisory Council***. The purpose is to review and provide input for current and future clinical information systems. Its responsibilities don't require a hospital-level council; therefore, it's a system-level council only with representation from all hospitals.

***CNO Council***. The purpose is to provide a voice for nursing leadership at the CNO and director levels, including additional members to ensure nursing leadership representation across the system. This council provides approval of senior-level nursing decisions as appropriate and provides support for the structure, process, and outcomes of Roper St. Francis Healthcare (RSFH) Professional Governance as indicated in the model (see Figure 4).

Every Wednesday, a system-level council (not including the CNO Council) met to promote consistency and engagement in these councils. One reason for inefficiency in decision-making in the previous model was the need to go to four different hospital councils for system decision-making. For example, if a nursing practice change was proposed, the proposal would have to go to each hospital's practice council for approval. The councils met at all different times during the month, and if one council had a suggestion for a change, the process would repeat. The process was very inefficient and led to decisions being made outside of shared governance.

Therefore, one of the biggest changes with the new professional governance structure was to the Quality and Practice Council and the Professional Engagement and Advancement Council. Recognizing that decisions in these councils must be made at the system level, yet wanting to maintain ownership at the hospital levels, the councils began meeting first as a hospital council for 1 hour and then joining the other hospital councils virtually for decision-making. This structure provides one place for issues and decisions to be presented rather than four different councils. Incorporating the system council into the hospital council meeting increased clinical nurses' engagement because it didn't require additional meeting time.

## IMPACT

The Verran Professional Governance Scale (VPGS) was used to measure the impact of the planned transition to the new professional governance structure. This 22-item survey uses a 7-point Likert-type scale for three domains of professional governance: professional obligation, collateral relationships, and decision-making.^8^

The presurvey began in November 2022 and was collected until January 2023. Results showed the organization had strong perceptions of professional governance prior to the restructuring intervention, reflecting the maturity needed for change. However, the participation rate ranged from only 22% to 29% of eligible direct care nurses.

The postsurvey was completed in October 2023, 10 months after the restructuring. There was a drastic improvement in the participation rate in the postsurvey, ranging from 33% to 48% of eligible nurses completing the postsurvey, with an increased participation rate of 17% to 118% across the system (see Table 2). This increase in the participation rate alone shows the improved engagement of direct care nurses and the impact of the revised professional governance structure in engaging clinical nurses. In addition to the higher postsurvey response rate, the means improved for the total composite and each subscale after the intervention; these results will be published after further analysis is completed. Although the initial Lean quality improvement project didn't require institutional review board (IRB) review, the institution's IRB approved the additional research study as exempt.

**TABLE 2:** Survey participation rates for direct care nurses

Hospital	Pre n (% eligible)	Post n (% eligible)	Increase in participation rate
A	119 (22)	216 (33)	50%
B	94 (25)	164 (42)	68%
C	31 (29)	52 (34)	17%
D	36 (22)	79 (48)	118%

In support of the business case for the project, the voluntary RN turnover for the system decreased from 20.29% at the end of 2022 to 17.62% at the end of 2023. On the RN engagement survey, the results for the question “I'm involved in decisions that affect my work” improved from 3.82 in 2022 to 3.93 in 2023. Overall patient satisfaction for the system for the “likelihood to recommend” question improved from 76.78% in 2022 to 78.47% in 2023. The system's total fall rate per 1,000 patient days decreased from 1.96 in 2022 to 1.74 in 2023. The system also saw a reduction in healthcare-associated infections, which many unit councils focused on in 2023. The Clinical and Research Excellence Council calculated the potential 2023 system cost savings related to nursing-led clinical inquiry initiatives and practice changes developed through the professional governance structure to be approximately $1.5 million.^9^-^11^ Based on these results, organizations that have a mature governance structure can successfully transition from shared to professional governance to improve nurse perceptions of professional obligation, collateral relationships, and decision-making.

### Specific examples of councils in action

A few specific examples of initiatives and decisions the councils made in 2023 that further demonstrate the impact of the change to professional governance include:

- *Quality and Practice Council*
  – The system-level Quality and Practice Council reviewed and approved 19 total nursing practice changes.
  – The council created a Bedside Shift Report (BSR) Task Force to explore the barriers to consistent BSR practice, create a standard of care that incorporated new BSR best practices, develop resources for supporting nurses and units in refocusing on consistent BSR, and determine consequences for nurses who choose not to practice BSR. This successful initiative improved patient-experience outcomes around BSR.
  – The council also approved and assisted with the implementation of a four eyes skin assessment initiative that reduced pressure injuries.
- *Professional Engagement and Advancement Council*
  – The newly revised system council implemented the first jointly planned Certified Nurses' Day and Nurses' Week for the system, which were previously handled at the hospital level.
- *Night Shift Council*
  – One hospital council created a nightly safety call via the system's messaging application, which resulted in a decrease in workplace violence incidents.
  – Another hospital council implemented a “quiet at night” initiative that resulted in improved patient experience outcomes.
  – All councils increased night-shift-specific events and recognition.

## IMPLICATIONS FOR NURSING LEADERSHIP

The nurse leader's role in providing resources, time, and support for engagement is essential to effective shared decision-making and is one of the main factors determining its success or failure. Nurse leaders must believe strongly in the importance of professional governance and work hard to ensure that resources and time are allotted for participation. Establishing professional governance engagement as an expectation for nurses and not as an obligation is one way to encourage more participation. Anecdotally, clinical nurses stated that most became engaged in councils because their nurse manager or another council leader asked them directly and provided them with encouragement and support. Additionally, having the CNO facilitate the Leadership Advisory Council signifies that the organization values professional governance.

The Nursing Excellence department and the Leadership Advisory Councils review the professional governance model and processes on a continuous basis. In 2024, two additional councils (Nurse Manager Council and Clinical Specialist Council) were requested by nurses in these roles and added to the model to provide an avenue for shared decision-making for these specific nursing roles. Overall, the performance improvement project to transition from shared to professional governance successfully led to increased clinical nurse engagement in councils at all levels (unit, hospital, and system) and more efficient council decision-making.
